# Glibenclamide, a Sur1-Trpm4 antagonist, does not improve outcome after collagenase-induced intracerebral hemorrhage

**DOI:** 10.1371/journal.pone.0215952

**Published:** 2019-05-01

**Authors:** Cassandra M. Wilkinson, Paul S. Brar, Celine J. Balay, Frederick Colbourne

**Affiliations:** 1 Department of Psychology, University of Alberta, Edmonton, Alberta, Canada; 2 Neuroscience and Mental Health Institute, University of Alberta, Edmonton, Alberta, Canada; Massachusetts General Hospital/Harvard Medical School, UNITED STATES

## Abstract

The sulfonylurea 1 transient receptor potential melastatin 4 (Sur1-Trpm4) receptor is selectively expressed after intracerebral hemorrhage (ICH). This upregulation contributes to increases in intracellular sodium. Water follows sodium through aquaporin channels, leading to cytotoxic edema. Even after edema is thought to have resolved, ionic dyshomeostasis persists, as does blood-brain barrier (BBB) damage. Glibenclamide, a hypoglycemic agent that inhibits Sur1-Trpm4, has been shown to reduce BBB damage and edema following infusion of autologous blood into the brain (ICH) as well as after other brain injuries. In order to further assess efficacy, we used the collagenase ICH model in rats to test whether glibenclamide reduces edema, attenuates ion dyshomeostasis, improves BBB damage, and reduces lesion volume. We tested a widely-used glibenclamide dose shown effective in other studies (10 μg/kg loading dose followed by 200 ng/hr for up to 7 days). Early initiation of glibenclamide did not significantly impact edema (72 hours), BBB permeability (72 hours), or lesion volume after ICH (28 days). Recovery from neurological impairments was also not improved by glibenclamide. These results suggest that glibenclamide will not improve outcome in ICH. However, the treatment appeared to be safe as there was no effect on bleeding or other physiological variables.

## Introduction

Intracerebral hemorrhage (ICH) is a devastating stroke with a 40% mortality rate [1]. In the hours after an ICH, ionic homeostasis becomes disrupted, and this dyshomeostasis can persist for weeks [2–4]. Sodium (Na) and chloride (Cl) concentration increase whereas potassium (K) concentration declines. These ionic perturbations are greatest near the hematoma, but extend well into the perihematoma zone [4]. Perhaps because of this, and other factors, there is considerable cellular injury (e.g., loss of dendrites [5]) and death in the perihematoma region [6]. Likely these ionic perturbations also directly impair neural function, and could lead to seizures, commonly seen in preclinical work and in patients [7,8]. Further indirect support comes from our research that shows that rehabilitation normalizes Cl levels in the peri-hematoma zone after experimental ICH [3], which might underlie how rehabilitation improves behavioral recovery. These data also suggest that pharmacological therapies to restore ion homeostasis may improve outcome after ICH.

Sulfonylurea receptor 1 (Sur 1) is constitutively expressed, but the transient receptor potential melastatin 4 (Trpm4) is not normally present in brain tissue. Sur 1 and Trpm4 are both upregulated and co-expressed as the heteromeric Sur1-Trpm4 channel after brain injury, such as ICH, ischemic stroke, and traumatic brain injury [9,10]. These channels allow for Na entry into cells, thereby contributing to cytotoxic edema [11] and likely persistent ionic dyshomeostasis. Glibenclamide, a Sur1 receptor antagonist, is being explored as a treatment to reduce edema after brain injuries, such as ischemic stroke, traumatic brain injury, and subarachnoid hemorrhage [12–15]. Higher doses of glibenclamide are used as a hypoglycemic agent to treat diabetes, as glibenclamide inhibits Sur1 receptors on pancreatic β cells, stimulating insulin release [13]. In ICH, lower doses of glibenclamide are a promising therapeutic due to the role Sur1-Trpm4 channels may play in edema formation and ion dyshomeostasis.

Two studies have explored whether glibenclamide improves outcome in a preclinical ICH model. Jiang et al. used the autologous whole blood model of ICH in Sprague Dawley rats, and found glibenclamide reduced edema, protected blood-brain barrier (BBB) integrity, and improved long-term neurological deficits [16]. Another study, using the collagenase model of ICH in rats, reported that glibenclamide reduced oxidative stress, inhibited apoptosis, and improved neurological deficits [17]. Neither of these studies measured ion concentrations nor did they assess lesion size. As lesion volume is a key predictor in patient and rodent outcomes, it makes sense to determine the impact of potential therapies on total lesion size [18,19], which is the definitive method to measure neuroprotection. Glibenclamide after ICH has been investigated clinically as well. Ghasami et al. compared the use of glibenclamide and insulin when given to diabetic hemorrhagic stroke patients [20]. The glibenclamide group had no benefit as compared to the insulin group. However, we note that this was a small, non-randomized, non-placebo-controlled trial, and further clinical work in hemorrhage would be needed.

In our study, we rigorously tested the effectiveness of glibenclamide after ICH in rats. We produced ICH using an intra-striatal injection of collagenase. As we were investigating the ability of glibenclamide to reduce edema and improve BBB integrity, we used the collagenase model that causes more extensive edema and BBB damage than the autologous whole blood model [21]. Arguably, the collagenase model may better represent the amount of edema and BBB injury seen in many ICH patients [4,21–23]. Further, ion dyshomeostasis persists for at least 14 days in the collagenase model [3], likely far longer than what occurs in the standard autologous whole blood model of ICH [2]. The effect of glibenclamide on edema, BBB integrity, and ion homeostasis have only been assessed using the autologous whole blood model of ICH. The use of multiple models is a recommended step in pre-clinical translational research [21,24,25], as ICH patients have heterogeneous injuries that are not reproduced by any one model. First, we assessed the safety of glibenclamide by measuring its effects on bleeding, blood glucose, core temperature, and activity. Glibenclamide affects vasodilation and could potentially impact bleeding after ICH [26]. These cardiovascular actions further support investigation using the collagenase model, which directly causes bleeding. Additionally, the effect of glibenclamide on core temperature has not been assessed, although it is needed, as core temperature can confound preclinical research and affect outcome in diverse ways [25,27]. Next, we determined the acute effects of glibenclamide on edema, BBB integrity, ion concentrations, neurological deficits, and blood glucose to directly assess the intended effects of blocking Sur1-Trpm4. Finally, we evaluated the long-term effects of glibenclamide on skilled reaching, walking ability, blood glucose, and lesion volume.

## Materials and methods

### Subjects

Procedures were in accordance with the Canadian Council on Animal Care Guidelines and were approved by the Biosciences Animal Care and Use Committee at the University of Alberta. All surgical procedures were performed under isoflurane anesthesia, and bupivacaine hydrochloride was used as an analgesic to minimize suffering.

We used 80 male Sprague Dawley rats (275–450 g, ~2–4 months old) from Charles River (Saint Constant, Quebec). Purina rodent chow and water were provided *ad libitum*, except when behavioural testing required food deprivation. Animals were single-housed, except during experiment 3, where they were housed in groups of 4, in a temperature- and humidity-controlled room with a 12-hour light cycle. All procedures were done in the light phase.

### Experimental design

In all experiments, animals were randomly assigned to groups and data was collected and analysed while blinded to group assignment. A complete experimental plan, including power calculations and statistical analyses were made prior to the start of the experiment and is available at https://www.ualberta.ca/science/about-us/contact-us/faculty-directory/fred-colbourne. *A priori* power analyses were based on expected effect size and variability of the primary endpoint.

In experiment 1, we assessed the impact of glibenclamide on bleeding, blood glucose, core temperature, and activity at 24 hours post-ICH to determine the safety of glibenclamide with the collagenase ICH model. Rats were randomized to either glibenclamide (n = 6) or vehicle (n = 6), which was estimated to give 80% power to detect a 40% change in hematoma volume. Note that this experiment was stopped after a blinded and planned interim analysis (at a N of 6 per group, set at α = 0.1) showed no change in hematoma volume.

In experiment 2, we determined the effect of glibenclamide on edema, BBB permeability, ion concentrations, blood glucose, and neurological deficits at 3 days post-ICH. With 16 animals per group (glibenclamide vs. vehicle), we anticipated having 80% power to detect a 1% increase or decrease in edema. This effect size was based on Jiang et al, who saw a 1.04% decrease in edema after treatment with glibenclamide [16].

In experiment 3, we assessed whether glibenclamide provided long-term efficacy by assessing skilled reaching, walking ability, and lesion volume at 28 days post-ICH. Animals were randomized to either glibenclamide (n = 18) or vehicle (n = 18), which was expected to give 80% power to detect a 30% increase or decrease in lesion volume. This effect size is detectable [19], and should be biologically meaningful.

### Telemetry Probe Implantation (Exp. 1)

Animals were anesthetized with isoflurane (4% induction, 2–2.5% maintenance, 60% N_2_O, and remainder O_2_) [28–30]. A sterile probe was inserted into the abdomen (Model TA10TA-F40, Data Sciences International, St. Paul, MN, accurate to ±0.1°C). Meloxicam (0.2 mg/kg SC) and bupivacaine hydrochloride (0.5 mg/kg SC) were administered for analgesia. Baseline data was recorded for 24 hours prior to ICH induction. Core temperature and activity were recorded every 30 seconds and averaged every hour. Post-ICH core temperature and activity readings were corrected hourly to baseline values in order to control for circadian rhythm effects. According to our typical analysis methods, extreme data points (e.g., above 42°C), which occasionally occur due to signal noise, were excluded.

### Blood glucose measurement

Blood glucose was measured using a glucometer (Contour Next One, Ascensia Diabetes Care, Mississauga, ON) in free feeding rats [29]. Immediately following induction of isoflurane anesthesia, the tail was pricked with a needle to obtain a small drop of capillary blood for analysis.

### Intracerebral hemorrhage

Rats were anesthetized (isoflurane) and rectal temperature was maintained at 37°C using a rectal thermometer probe and a heating pad. Bacterial collagenase (Type IV-S, Sigma, 1.0 μL of 0.6 U/μL in saline) was infused into the striatum at 0.5 mm anterior, 3.5 mm lateral, and 6.5 mm down from Bregma over 5 minutes [28,31,32]. The needle was left in place over 5 minutes to prevent backflow. A screw was used to close the needle hole and the incision was stapled shut. Bupivacaine hydrochloride was applied subcutaneously for analgesia. Collagenase was injected into the left striatum, except in experiment 3, where collagenase was injected into the side contralateral to the preferred paw, as assessed by the skilled reaching task baseline. Animals were randomly assigned following the ICH procedure.

### Glibenclamide administration

Glibenclamide (Abcam, Product # ab120267, Toronto, ON) was prepared using dimethylsufoxide, sodium hydroxide, and saline as described [12]. Glibenclamide was infused subcutaneously starting at 2 hours post-ICH using a mini-osmotic pump (Alzet osmotic pumps, 2001, 1.0 μl/h) to give a dose of 200ng/hour. This was the same dose used in previous work in hemorrhage and ischemia [12,16,33]. Mini-osmotic pumps were primed overnight before implant. At the time of pump insertion, a loading dose of 10 μg/kg was given intraperitoneally. Drug delivery was verified by measuring amount of solution remaining after pump removal.

### Hemoglobin assay (Exp. 1)

A spectroscopic hemoglobin assay was used to determine the amount of hemoglobin in each hemisphere based upon a standard curve [21]. Hematoma volume, our primary endpoint in experiment 1, was calculated as: ipsilateral blood volume minus contralateral blood volume, which accounts for blood in the vasculature not attributed to the hematoma.

### Behavioural assessment

The Montoya staircase task was used to measure skilled reaching ability and was the primary behavioural endpoint [19]. During training and testing, animals were maintained at 90% of their free feeding weight to encourage reaching. Rats were trained twice daily, 5 days per week for 4 weeks prior to ICH. The average number of pellets retrieved by the dominant paw over the last 3 training days was used as the baseline score. Animals were excluded if they failed to retrieve at least 9 out of 21 pellets (45 mg reward pellets, Bio-Serv Flemington, NJ). Excluded animals still underwent behavioural testing and were otherwise included in the experiment. Testing took place on days 8–10 and 25–27 post-ICH.

Walking ability was assessed using the horizontal-ladder walking test [19]. Rats were videotaped while crossing a horizontal ladder with variable spaced rungs (3–5 cm apart). On each testing day, 2 crosses were recorded. The number of slips for each limb was recorded and averaged across the trials. Animals were assessed at baseline (pre-ICH), and days 7 and 28 post-ICH. Rats were excluded from only the ladder test if they failed to cross the apparatus twice in the baseline testing session.

Neurological deficits were assessed using the Neurological Deficit Scale (NDS), which is comprised of beam walking, spontaneous circling, forelimb flexion, bilateral forelimb grasping, and contralateral hind limb retraction [19]. Scores ranged from 0–14, with 0 indicating no impairments.

### Brain water and ion determination (Exp. 2)

Rats were briefly anesthetized (isoflurane) and received an injection of Magnevist (gadopentetate dimeglumine; 2.5 mL/kg; Bayer, Mississauga, ON) into the tail vein [4]. Gadolinium (Gd) does not normally cross an intact BBB, and thus high Gd content in the brain is an indicator of BBB permeability [34]. Magnevist has a short half-life, so rats were decapitated 10 minutes after injection. Brains were rapidly removed and assessed for water and element content.

Brain water content was measured using the wet weight-dry weight method, our primary endpoint in experiment 2 [35]. Here, we define edema as increased water content, and note that this can include serum extrusion as well as edema. Brains were blocked from 2 mm anterior to 4 mm posterior to the collagenase needle tract, and tissue was separated into striatal and cortical tissue for each hemisphere. The cerebellum was taken as a control. The tissue wet weight was measured, and the dry weight was determined after tissue samples were baked at 100°C for 24 hours.

After measurement of brain water content, the dried tissue was digested in high purity nitric acid (Sigma-Aldrich) for 1 week, as done previously [4,28]. Na, K, iron (Fe), and Gd were measured using inductively coupled plasma mass spectrometry (ICP-MS; Thermo Scientific ICAP-Q quadrupole ICP-MS, Canadian Centre for Isotopic Microanalysis, University of Alberta). Fe was used as a rough indicator of hematoma volume. Other elements measured but not presented include magnesium, phosphorus, calcium, manganese, copper, zinc, and selenium (data available in S1 Dataset).

### Histology (Exp. 3)

On day 28 post-ICH, animals were injected with 100 mg/kg IP of Na pentobarbital and perfused with 0.9% saline followed by 10% neutral-buffered formalin. Brains were cryoprotected with 20% sucrose prior to cryostat sectioning. Coronal sections were 40-μm thick and taken every 200 μm from before, through, and beyond the lesion. Tissue was stained with cresyl violet and lesion volume was analyzed, as previously done [19]. The volume of a hemisphere was calculated as: (average area of complete coronal section of hemisphere-area of damage-area of ventricle) x interval between sections x number of sections). The volume of tissue lost, which includes observable tissue loss, atrophy and ventriculomegaly, was calculated as the contralateral hemisphere minus the ipsilateral hemisphere volume. Total tissue loss was the primary endpoint of experiment 3, and *a priori*, what we considered to be our most important endpoint overall.

### Statistical analysis

Data were analyzed using GraphPad Prism (v. 6.0, GraphPad Software Inc., La Jolla, CA) or RStudio (v. 1.1.456, RStudio, Boston, MA). All data are presented as mean ± 95% confidence interval, except non-parametric data, which are expressed as median ± interquartile range. Two group comparisons were made using a t-test, except NDS, which was compared with a Mann-Whitney U-test. Repeated measures data was assessed using a 2-way ANOVA with Sidak’s multiple comparisons test. Level of statistical significance was set at α = 0.05.

## Results

### Mortality and exclusions

In experiment 1, 1 rat died due to surgical complications during the telemetry probe implantation, prior to group assignment. In experiment 2, 1 animal in the glibenclamide group and 3 animals in the vehicle group were excluded from Gd analysis due to technical problems with the Gd injection. These animals were excluded at the time of the procedure, prior to data analysis. In experiment 3, 1 animal from the vehicle group was excluded from skilled reaching analysis due to failure to meet baseline criteria. One glucose reading in the vehicle group was excluded due to failure to obtain sufficient blood sample. Five individual ladder videos were excluded from analysis due to experimenter error.

### Experiment 1

#### Temperature and activity

**Fig 1:**
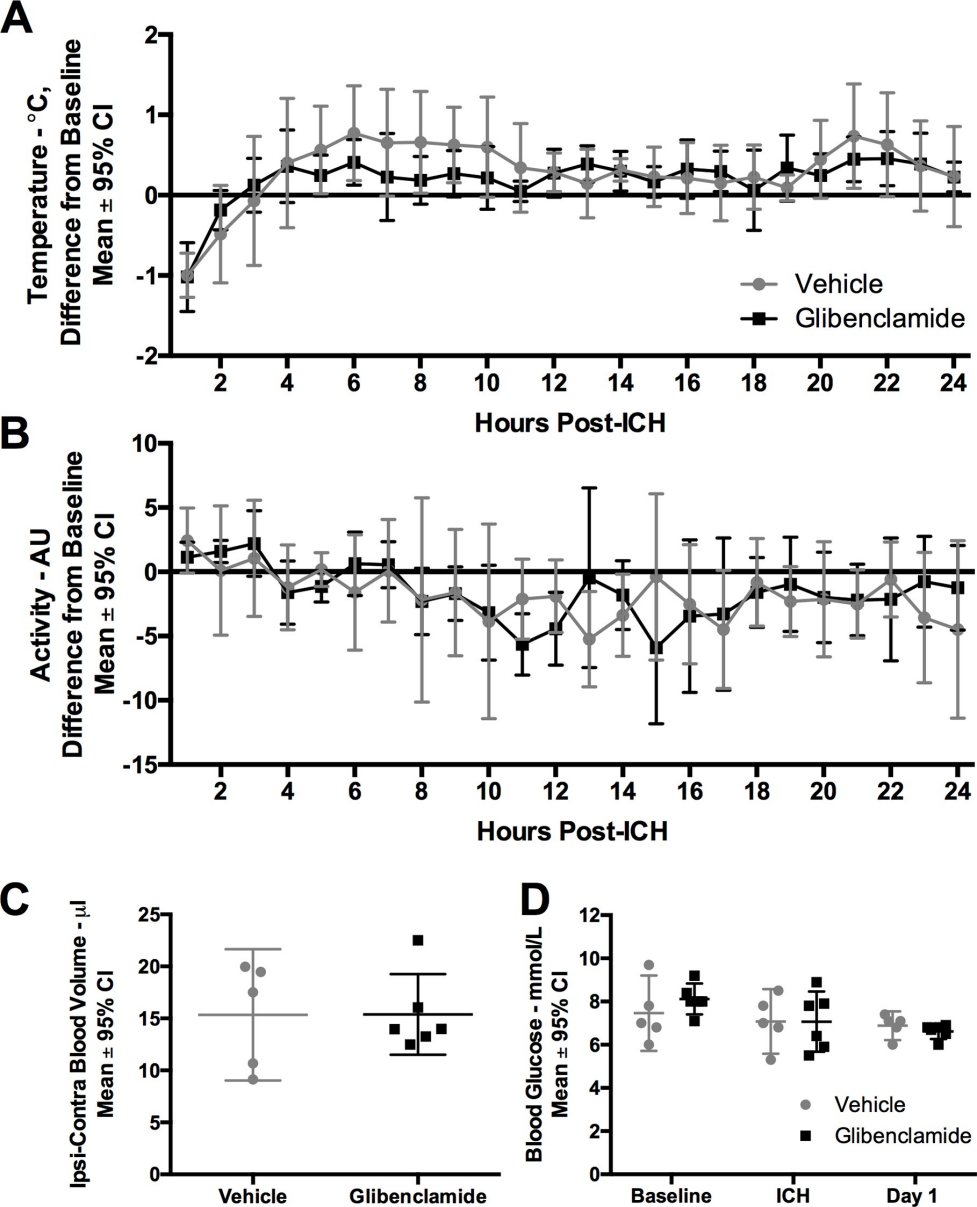
Experiment 1. Glibenclamide did not significantly impact (A) core temperature (*P* = 0.187, group main effect) or (B) activity (*P* = 0.876, group main effect) after ICH (n = 6 in glibenclamide group, n = 5 in control group). Core temperature (°C) and activity (AU, arbitrary units–detections of animal movement over a receiver [36]) data were taken using implanted telemetry devices. Core temperature and activity averages for each hour were corrected for the same hour of baseline data to account for time of day effects (e.g., circadian rhythms). Rectal temperature was regulated during surgical procedures; the core temperature drop within the first few hours was due to rapid but mild cooling after post-surgical anaesthesia. (C) Bleeding (*P* = 0.991) and (D) blood glucose (*P* = 0.763, group main effect) were both unaffected by glibenclamide. Sample sizes were n = 6 in glibenclamide group, n = 5 in control group.

Core temperature varied over time after ICH, with core temperatures above baseline until approximately 12 hours post-ICH, indicating mild post-stroke hyperthermia (Fig 1A, *P* < 0.001, time main effect, partial η^2^ = 0.901, large effect). Core temperature was not significantly impacted by glibenclamide (Fig 1A, *P* = 0.187, group main effect; *P* = 0.323, interaction effect). The ICH reduced spontaneous activity, (Fig 1B, *P* = 0.001, time main effect, partial η^2^ = 0.683, large effect), but glibenclamide did not impact activity levels (Fig 1B, *P* = 0.876, group main effect, *P* = 0.240, interaction effect).

#### Hematoma volume

Glibenclamide did not significantly alter hematoma volume measured 24 hours post-ICH (Fig 1C, *P* = 0.991).

#### Blood glucose

Blood glucose values dropped at 24 hours after ICH (Fig 1D, *P* = 0.040, time main effect, partial η^2^ = 0.795, large effect). This was likely due to rats eating less. Glibenclamide did not significantly impact blood glucose (Fig 1D, *P* = 0.763, group main effect; *P* = 0.475, interaction effect).

### Experiment 2

#### Brain water content

**Fig 2:**
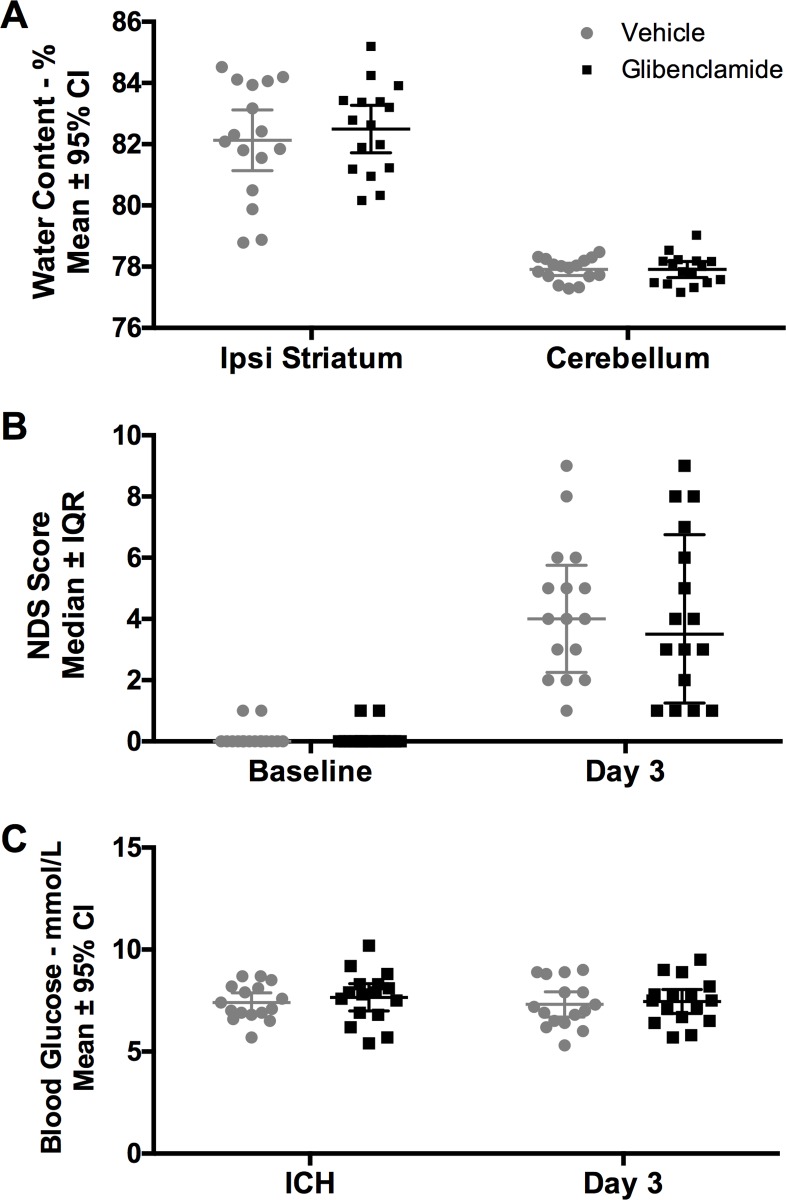
Experiment 2. Glibenclamide did not reduce (A) brain water content (*P* = 0.629, group main effect) or (B) neurological deficits (*P* = 0.743). (C) Blood glucose was not impacted by glibenclamide (*P* = 0.531, group main effect). Sample size was n = 16/group.

Water content was increased in the ipsilateral striatum after ICH (Fig 2A, *P* < 0.001, region main effect; Cohen’s *d* = 2.68). Glibenclamide did not affect the amount of water in the brain (Fig 2A, *P* = 0.629, group main effect; *P* = 0.897, interaction effect). There was no effect of ICH or glibenclamide on water content in the contralateral hemisphere (see S1 Dataset).

#### NDS

The ICH led to significant neurological deficits (Fig 2B, *P* < 0.001, Cohen’s *d* = 2.31, large effect), which were unaffected by glibenclamide (Fig 2B, *P* = 0.743).

#### Blood glucose

There was no effect of glibenclamide or time on blood glucose concentration (Fig 2C, *P* = 0.506, time main effect; *P* = 0.531, group main effect; *P* = 0.823).

#### Ion concentrations

**Fig 3:**
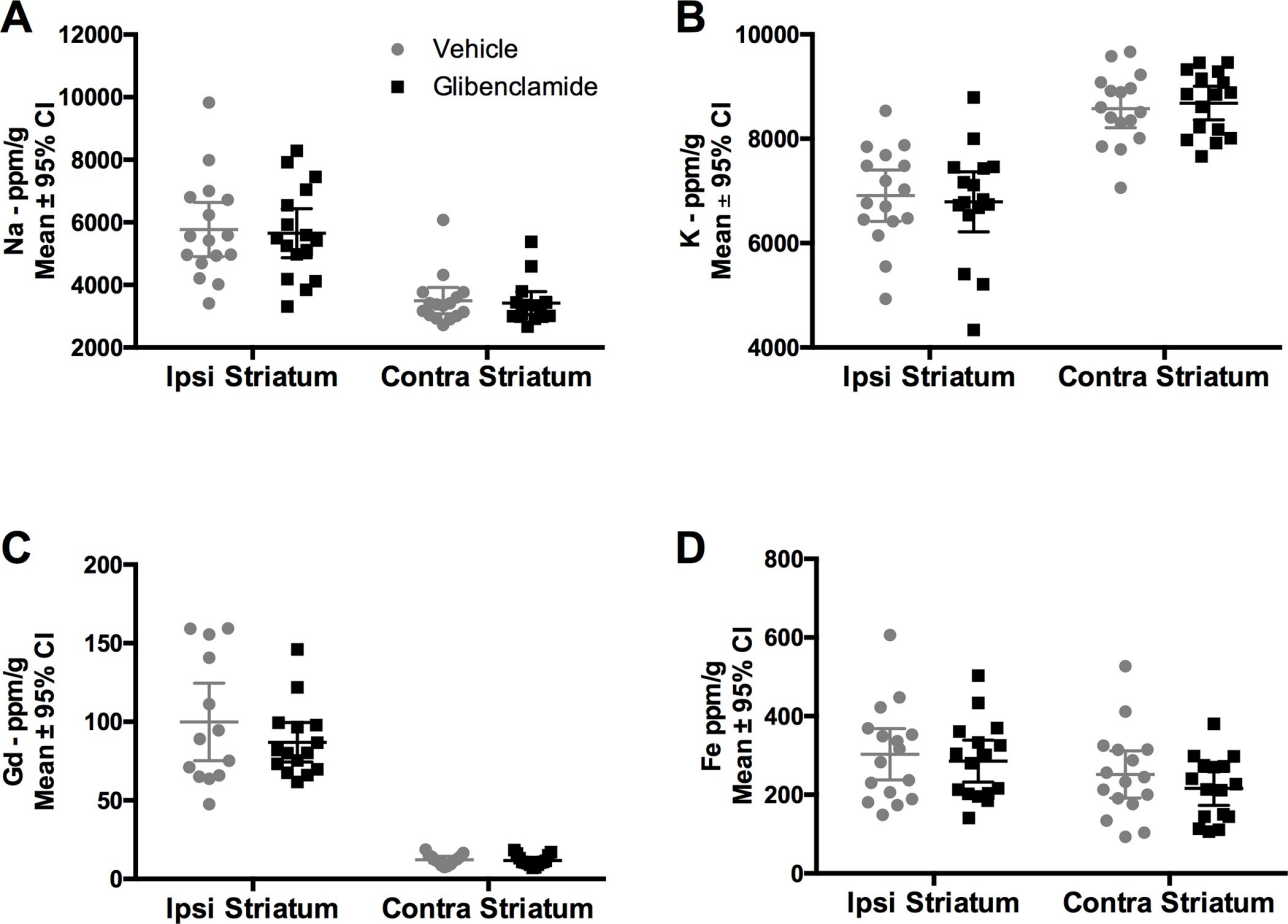
Experiment 2-Ion Concentrations. (A) Na (*P* = 0.798) and (B) K (*P* = 0.979) concentrations were not affected by glibenclamide, despite being increased in the hemisphere ipsilateral to the ICH. Treatment with glibenclamide did not impact (C) BBB permeability, as assessed with Gd (*P* = 0.288), or amount of Fe in the striatum (*P* = 0.317, group main effect). Sample size was n = 16/group.

The integrity of the BBB was measured using tissue Gd content (ICP-MS). While ICH increased Gd levels significantly (Fig 3C, *P* < 0.001, Cohen’s *d* = 0.50, medium effect), glibenclamide did not significantly affect Gd concentration (Fig 3C, *P* = 0.288). Glibenclamide did impact the distribution of Gd concentrations in the ipsilateral striatum (*P* = 0.031, Shapiro-Wilk normality test), as data in the glibenclamide group was positively skewed (skewness = 1.47), indicating fewer values with higher levels of Gd. Thus, we also analyzed the Gd data using a non-parametric test which also indicated glibenclamide did not affect BBB permeability (*P* = 0.777). After ICH, Na concentration was significantly increased (Fig 3A *P* < 0.001, Cohen’s *d* = 1.94, large effect), but not significantly altered by glibenclamide (Fig 3A, *P* = 0.798). K was lowered after ICH (Fig 3B, *P* < 0.001, Cohen’s *d* = 2.11, large effect), but glibenclamide did not attenuate this decrease (Fig 3B, *P* = 0.979). ICH increased Fe concentration in the ipsilateral hemisphere (Fig 3D, *P* = 0.025, region main effect). Glibenclamide did not significantly change Fe concentration (Fig 3D, *P* = 0.317, group main effect; *P* = 0.735, interaction effect), which indicates that the hematoma size and resolution did not differ between groups.

**Fig 4:**
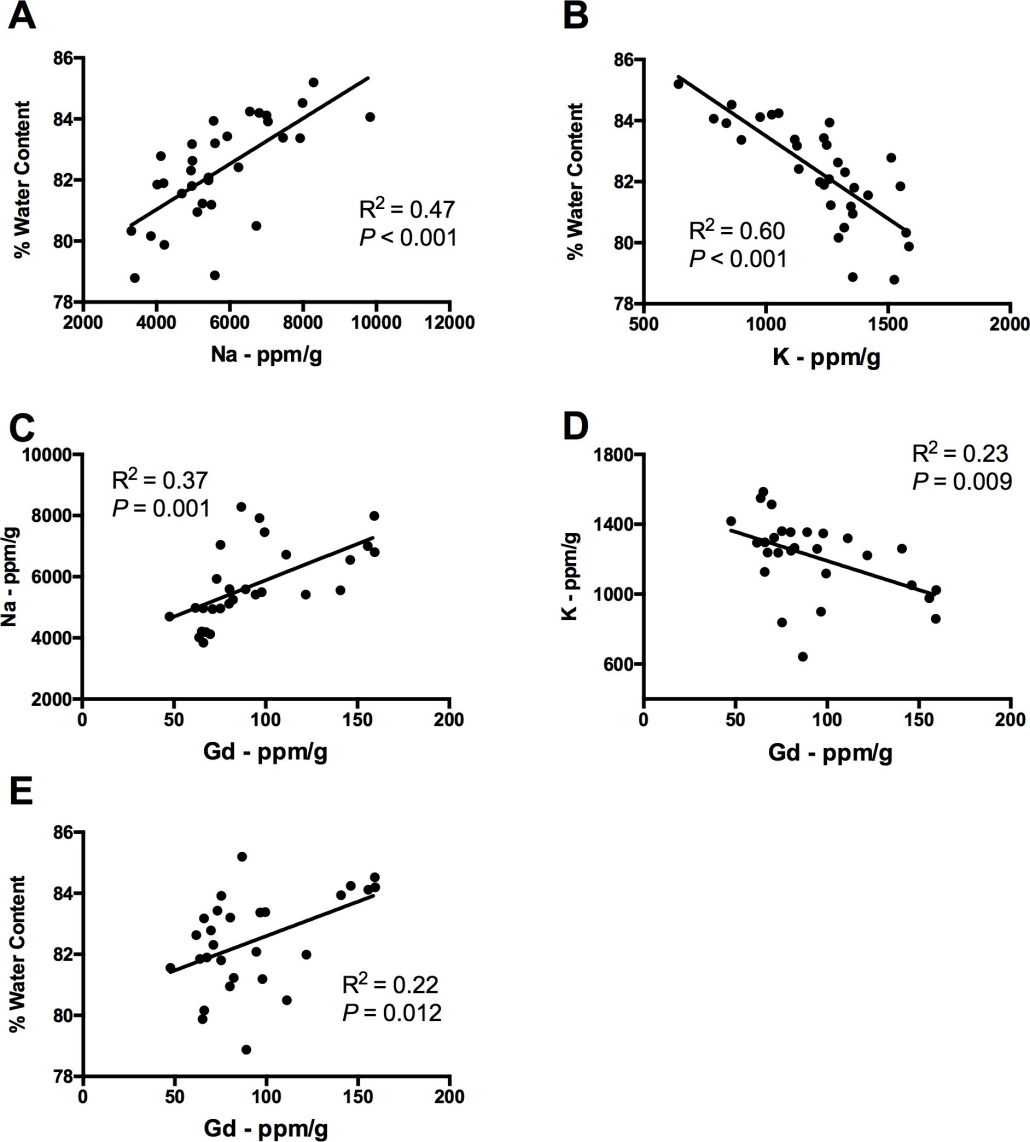
Experiment 2-Correlations. (A) Increased Na (R^2^ = 0.47, *P* < 0.001), and (B) decreased K (R^2^ = 0.60, *P* < 0.001) was associated with increased ipsilateral striatum edema. (C) More Na (R^2^ = 0.37, *P* = 0.001) and (D) less K (R^2^ = 0.23, *P* = 0.009) was related to greater amount of Gd extravasation. (E) More Gd extravasation was positively associated with edema (R^2^ = 0.22, *P* = 0.012).

Ion concentrations (Na, K, and Gd) and edema levels were closely related. Increased Na was associated with increased edema (Fig 4A, R^2^ = 0.47, *P* < 0.001), and decreased K was associated with increased edema (Fig 4B, R^2^ = 0.60, *P* < 0.001). Similarly, a larger increase in Na predicted a greater amount of BBB permeability (Fig 4C, R^2^ = 0.37, *P* = 0.001), as did a larger decrease in K (Fig 4D, R^2^ = 0.23, *P* = 0.009). A greater extent of BBB permeability, as assessed with Gd, predicted increases in edema (Fig 4E, R^2^ = 0.22, *P* = 0.012).

### Experiment 3

#### Lesion volume

**Fig 5:**
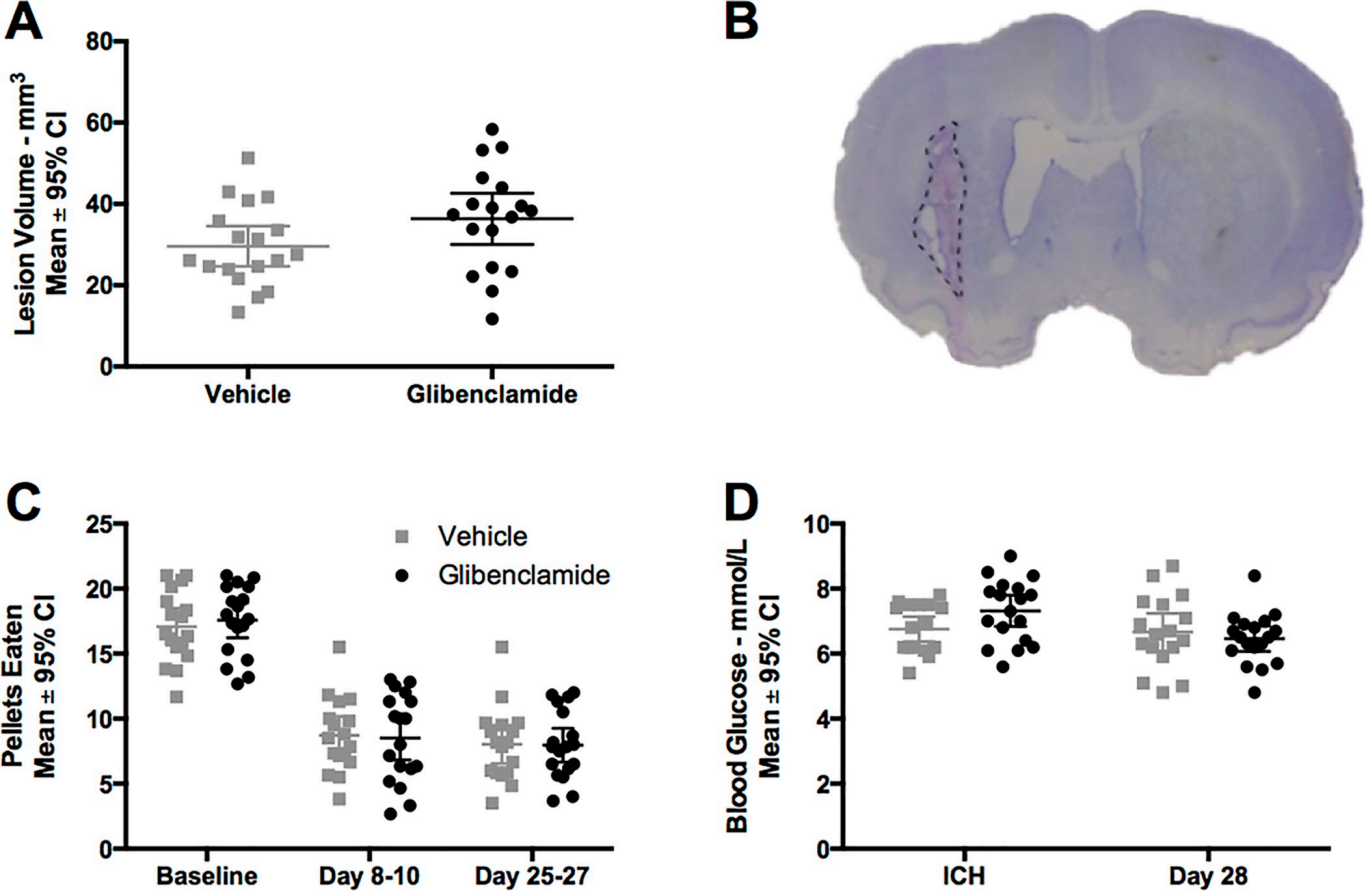
Experiment 3. Glibenclamide did not affect (A) tissue lost at a 28-day survival (*P =* 0.089). (B) Representative lesion volume image (vehicle group) demonstrating area of dead tissue (dashed line) and ventriculomegaly (larger ventricle). (C) reaching ability (*P* = 0.927, group main effect) and (D) blood glucose (*P* = 0.419, group main effect) we not impacted by glibenclamide. Sample size was n = 18/group.

Collagenase infusion caused significant damage to the striatum and ventriculomegaly (Fig 5B). Glibenclamide increased tissue lost by 23% at 28 days post-ICH, but this was not statistically significant. (Fig 5A, *P =* 0.089, Cohen’s *d* = 0.56).

#### Behaviour

**Fig 6:**
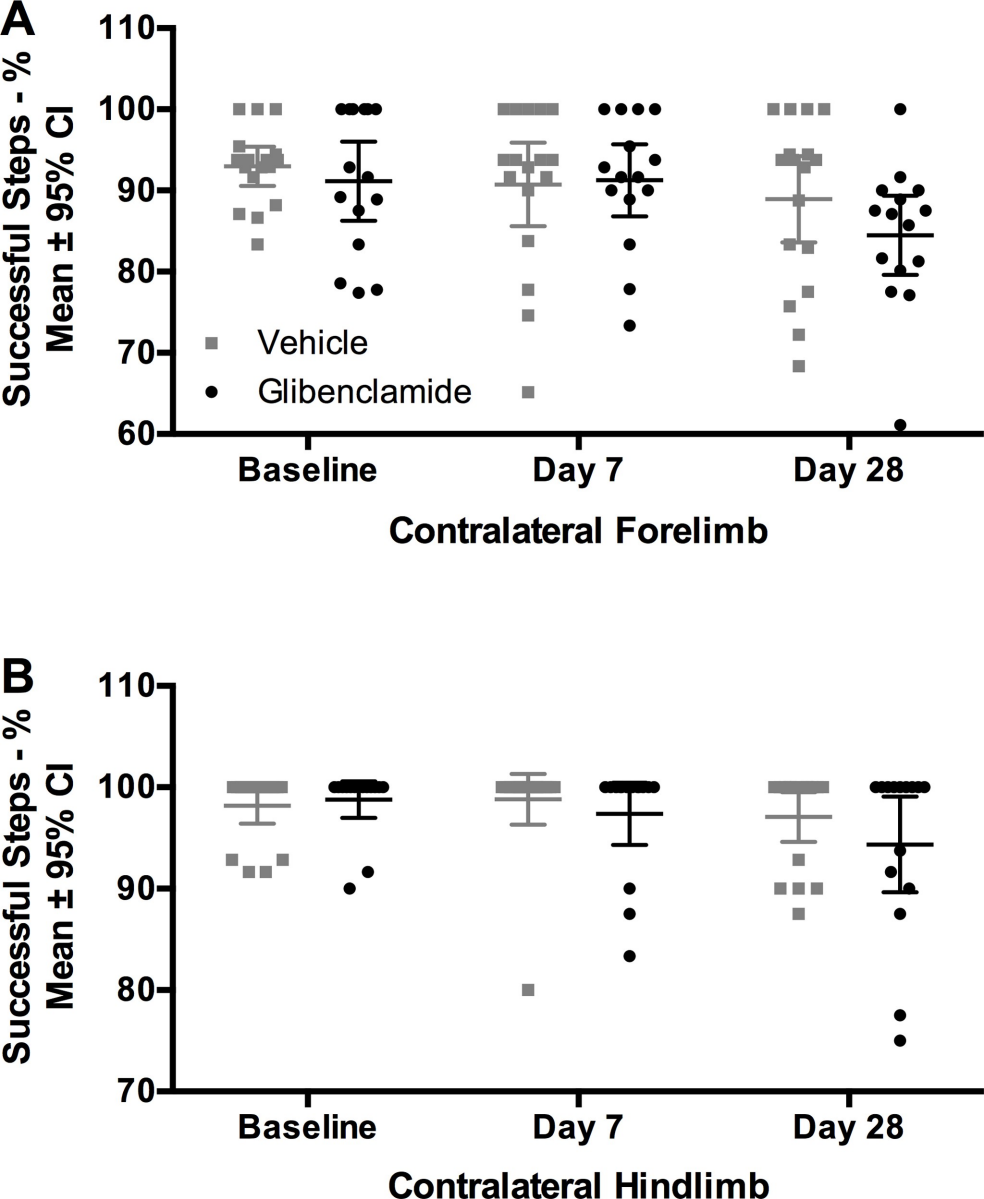
Experiment 3-Ladder. After ICH, walking ability was impaired (all *P<*0.020). Glibenclamide did not impact walking ability in (A) the contralateral forelimb or (B) the contralateral hindlimb (all *P>*0.11). Sample size was n = 18/group. Data from uninjured limbs available in S1 Dataset.

As expected, ICH caused reaching impairments in the staircase test. (Fig 5C, *P* < 0.001, time main effect, partial η^2^ = 0.994, large effect). Glibenclamide did not attenuate the impairments in reaching ability (Fig 5C, *P* = 0.927, group main effect; *P* = 0.798, interaction effect). Similarly, ICH caused significant impairments in walking ability in both forelimbs and the impaired hindlimb (Fig 6, all *P<*0.020, all partial η^2^ between 0.850 and 0.881, large effect). After ICH, the unimpaired hindlimb was not significantly affected (*P =* 0.638, partial η^2^ = 0.472). There was no effect of glibenclamide on walking ability with any limb (Fig 6, all *P>*0.110). Unimpaired limb data is not shown but is available in S1 Dataset.

#### Blood glucose

Glucose levels dropped post-ICH (Fig 5D, *P* = 0.035, time main effect, partial η^2^ = 0.829, large effect), and glibenclamide had no effect (Fig 5D, *P* = 0.419, group main effect; *P* = 0.086, interaction effect).

## Discussion

Glibenclamide, a Sur1-Trpm4 inhibitor and hypoglycemic agent, has shown promising results in ischemic and hemorrhagic stroke, including reducing edema, mortality, and functional deficits [12,14,16,17]. In contrast to those findings, glibenclamide did not reduce BBB permeability, element concentration alterations, edema, behavioral impairment or brain injury after collagenase-induced ICH in our experiments. The failure to affect BBB injury, ion alterations and edema suggests that glibenclamide will not mitigate brain swelling or intracranial pressure rises after ICH in patients. Furthermore, the lack of benefit against functional impairments and brain injury after ICH does not support the clinical use of glibenclamide as a neuroprotectant after ICH. Fortunately, physiological measures, including core temperature, activity, and blood glucose were not affected by glibenclamide. As well, hematoma volume was not affected.

Many preclinical glibenclamide studies, which support clinical investigation, have focused on reductions in edema [10,13,37,38]. Similarly, a previous study in ICH found that the same dose of glibenclamide used here significantly reduced brain water content [16], while we report no change in water content. This discrepancy is hard to reconcile because Zhou et al. used the autologous whole blood model, where most of the increase in brain water content arises from serum extrusion and not true edema [21]. Conversely, the collagenase model causes considerably more extensive BBB damage and edema [21], which one would think would be more amenable to glibenclamide. Perhaps the extent of injury after even moderate collagenase ICH is still too devastating, and despite blocking an important transporter, ions and water can still move freely through the damaged BBB. The autologous whole blood model has considerably less BBB damage [21], and thus simply blocking the Sur1-Trpm4 transporter may be an effective strategy in this environment. The magnitude of injury and edema observed in our study was modest, and caused no mortality. In a model of traumatic brain injury, which is also characterized by extensive BBB damage [39], it was also shown that a similar dose of glibenclamide did not attenuate increases in edema [40]. Therefore, the failure of glibenclamide was likely not because the ICH was exceptionally severe, but more so that the nature of damage in collagenase ICH is too disruptive or complex.

While our findings suggest that glibenclamide is safe to use, further testing should be conducted. Glibenclamide is currently being clinically investigated for use after large hemispheric infarction, an injury associated with hemorrhagic transformation [41]. Given the likelihood of hemorrhagic transformation in this population, the safety of glibenclamide after hemorrhage must be considered. Our data suggests no increase in bleeding, edema, or BBB damage. However, we saw a non-significant increase in lesion volume (Cohen’s *d* = 0.56, moderate effect size) in the glibenclamide group. This suggests that glibenclamide may worsen injury after hemorrhage, but we note that we are underpowered to detect an effect of this size. Unfortunately, other ICH studies did not examine lesion size, so we do not know if this effect occurred in those experiments [16,17]. No other study of glibenclamide in ICH has quantified lesion volume or total tissue loss (including cell death, cavity formation, and ventriculomegaly), which is an accepted and unambiguous measure of neuroprotection [25].

With our novel approach, we were able to measure edema, BBB permeability, and ion concentrations in the same tissue. ICP-MS enables precise determination of element concentrations, including Gd, Na, K, Fe, and others, excluding chloride. This measure of BBB permeability has translational relevance, as Magnevist is used in clinical imaging [34]. Further, assessing Gd extravasation in dried tissue assesses the amount of BBB permeability while excluding edema confounds, which would affect element concentrations on a per gram of wet weight basis [42]. Other common BBB permeability assays, such as Evans Blue, are unable to control for edema, and have been heavily criticized [43–45]. We are the first to directly assess the relationship between Na, K, edema, and BBB permeability in an experimental ICH model. The extent of Na increases and K decreases are closely related to increased edema, as reported by others [46,47]. Additionally, the amount BBB permeability correlates with both Na and K dyshomeostasis as well as edema. This data provides further support that edema, BBB permeability, and ionic dyshomeostasis have closely related mechanisms of damage after ICH [4,11,48], but the sometimes weak correlations suggest that other key mechanisms are involved. Interestingly, data using x-ray fluorescence imaging, a method used to precisely and spatially image elements in tissue, shows that BBB permeability and ionic dyshomeostasis are not spatially correlated [4].

The current study has limitations. We only assessed one dose of glibenclamide, and perhaps other doses may have conferred benefit. However, the dose we used is most commonly used after brain injury [12,16,33,49], as it selectively inhibits Sur1-Trpm4 and does not impact blood glucose levels. Additionally, the mini-osmotic pumps maintain a steady plasma concentration of glibenclamide, reducing risk of fluctuations in blood glucose. Further, we did not assess brain levels of glibenclamide to ensure the drug was biologically available in brain tissue. However, effects in the brain (e.g., lowering brain water content) with the same dose in other studies certainly suggests that this dose is bioavailable in the brain after injury [12,16,17,33]. We did not directly assess expression of Sur1 or Trpm4 in the collagenase model of ICH. However, Sur1-Trpm4 expression is reproducibly increased after ICH in rats, clinical ICH, and other preclinical models of injury such as ischemia and subarachnoid hemorrhage [9,10,13,16,37,38]. Glibenclamide may only work in large insults, and data in ischemia suggests glibenclamide can lower mortality by reducing life-threatening edema [14]. Our study had no mortality, and thus we may be unable to detect such benefits in ICH. However, we did see large (~4%), non-life-threatening increases in edema that glibenclamide did not reduce. We acknowledge that glibenclamide may reduce mortality in cases where edema is increased to a greater extent. Similarly, glibenclamide may improve outcome when using different models, such as comorbidities (e.g., diabetes, hypertension), aged animals, or female animals, which we did not test. While our studies were designed to have adequate power to detect biologically meaningful effects, it is possible as in all negative studies, that smaller effects went undetected.

We have taken steps to reduce bias and improve translation quality in this study. *A priori* experimental design with power calculations [50], statistical plan, and exclusion criteria reduce the potential for unconscious bias to interfere with the course of the study. *Post hoc* changes to experimental design or statistical analysis increase the rate of type I errors in a given study [51–53]. In this study, there were no *post hoc* changes made. Translational quality is another strength of this study. We used blinding, randomization, multiple endpoints, short- and long-term assessment, multiple measures of functional outcome, and a direct, unambiguous measure of neuroprotection [24].

In conclusion, this study demonstrates that glibenclamide is not beneficial after ICH. Glibenclamide failed to attenuate injury and improve outcome in every endpoint measured, including both the acute and chronic phases after ICH. Despite benefits seen in other pre-clinical ICH studies, we did not find any reduction in edema, BBB permeability, bleeding, or lesion volume after treatment with glibenclamide.

## Supporting information

S1 Dataset All data contained in this study. (XLSX)
